# The Impact of Sexualized Video Game Content and Cognitive Load on State Rape Myth Acceptance

**DOI:** 10.3389/fpsyg.2021.614502

**Published:** 2021-03-15

**Authors:** Tania Noël, Frank Larøi, Jonathan Burnay

**Affiliations:** ^1^Psychology and Neuroscience of Cognition Research Unit, Faculty of Psychology, Speech and Language Therapy, and Education, University of Liège, Liège, Belgium; ^2^Department of Biological and Medical Psychology, Faculty of Psychology, University of Bergen, Bergen, Norway; ^3^NORMENT – Norwegian Center of Excellence for Mental Disorders Research, University of Oslo, Oslo, Norway

**Keywords:** video games (psychology), sexualization, cognitive load, rape myths acceptance, humanness, attitude toward women

## Abstract

The potential negative impact of sexualized video games on attitudes toward women is an important issue. Studies that have examined this issue are rare and contain a number of limitations. Therefore, it largely remains unclear whether sexualized video games can have an impact on attitudes toward women. This study examined the consequences of sexualized video game content and cognitive load (moderator) on rape victim blame and rape perpetrator blame (used as a proxy of rape myth acceptance), and whether the degree of humanness of the victim and of the perpetrator mediated these effects. Participants (*N* = 142) played a video game using sexualized or non-sexualized female characters. Cognitive load was manipulated by setting the difficulty level of the game to low or high. After gameplay, participants read a rape date story, and were then asked to judge the victim’s and the perpetrator’s degree of responsibility and humanness. Based on the General Aggression Model (GAM), it was hypothesized that playing the video game with a sexualized content would increase the responsibility assigned to the victim and diminish the responsibility assigned to the perpetrator. Further, degree of humanness of the victim and the perpetrator was expected to mediate this relation. The results were partially consistent with these predictions: Playing a video game containing sexualized female characters increased rape victim blame when cognitive load was high, but did not predict degree of humanness accorded to the victim. Concerning the perpetrator, video game sexualization did not influence responsibility, but partly influenced humanness. This study concludes that video games impact on attitudes toward women and this, in part, due to its interactive nature.

## Introduction

Despite the increase attention to violence against women through movements such as “#MeeToo” or Amnesty’s “No is no” campaign, sexual violence remains a serious problem (Amnesty International, 2019). It is regrettable that such violence is often trivialized. According to a survey from the European Commission (2016), more than 1 out of 10 people in the European Union believe that sexual intercourse without consent may be justified in certain circumstances, for example, if the victim (generally a woman) is voluntarily going home with someone, if she was drunk or under the influence of drugs, if she was wearing revealing clothes, or if she did not say “no” clearly (European Commission, 2016). One explanation of this trivialization of sexual violence is based on several stereotypes surrounding it and often subsumed under what is called rape myth (Buddie and Miller, 2001). Rape Myth Acceptance (RMA) involves any belief that minimizes the act of rape or leads to victim blame, that is, to hold the victim partially or fully responsible for being raped while minimizing/disregarding the responsibility of the perpetrator (Burt, 1980; Lonsway and Fitzgerald, 1994; Loughnan et al., 2013). Such beliefs vary but can be grouped globally into four main categories (Mcmahon and Farmer, 2011): having the attitude that (1) the woman provoked her own rape (e.g., the outfit she wore was judged as too sexualized or she acted too suggestively), (2) the rape was not really a rape (e.g., the woman did not fight back enough or was unclear when refusing the sexual act), (3) men are not responsible for a rape (e.g., men cannot control their sexual needs or may have been too drunk to understand that it was a rape) and (4) women can lie about the rape (e.g., she lied to protect herself or as an act of revenge).

In this study, we focused on video games as one possible contributing factor to the prevelance and believability of rape myths (i.e., the increasing of rape victim blame and the decreasing of rape perpetrator blame) for several reasons. Video games are a widespread activity among people of all ages, including adults. For example, in Europe, more than half of adults play video games, which equals to some 250 million gamers in the EU (Interactive Software Federation of Europe, 2019). It should be noted that in the majority of video games, female characters are objectified (i.e., treated like objects instead of humans) and also dehumanized (Burgess et al., 2007; Summers and Miller, 2014). Indeed, in video games, women are either a damsel in distress, a reward, or a sex object. Furthermore, one of the most common general characteristics is that female video game characters are often sexualized, i.e., depicted with large breasts and buttocks, small waists, and female characters show large amounts of exposed skin (Burgess et al., 2007; Downs and Smith, 2010; Summers and Miller, 2014; Lynch et al., 2016). Additionally, we know that gender-based and sexual violence not only remain a current problem in real life (Amnesty International, 2019), but that this problem also turns out to be very real in the specific context of online gaming. Research has shown that, while playing online, female gamers often report experiences of harassment, and other negative behaviors such as trash talk or discriminatory player interaction (Nakamura, 2012; Salter and Blodgett, 2012; Gray et al., 2017). More recently, the impact of sexualization in video games on online sexual harassment has even been demonstrated experimentally (Burnay et al., 2019). However, thus far, very few studies have examined the potential impact of sexualized video game characters on negative attitudes toward women. This study attempts to fill this gap in the literature.

**FIGURE 1:**
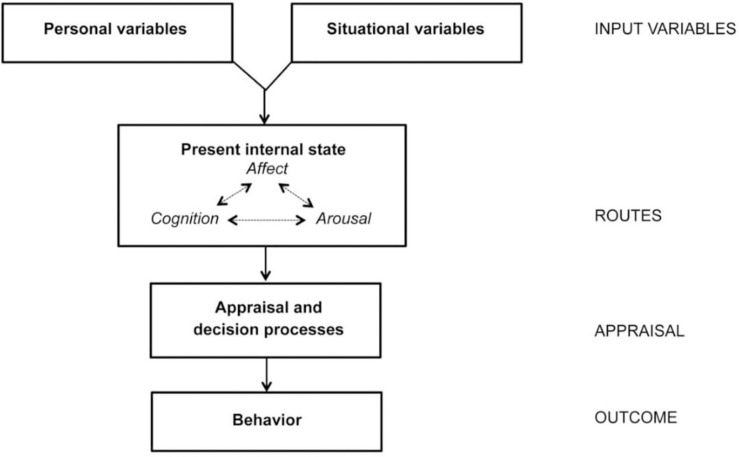
General aggression model (Bushman, 2017).

In order to explain the impact of sexualized video game content on the attitude toward women, the present study was based on the General Aggression Model (GAM; Bushman, 2017; Anderson and Bushman, 2018). Initially designed to explain the effect of violence exposure through the media, the GAM can be extended to exposure to sexualized video game content (Dill et al., 2008). According to the GAM, the development of aggressive behaviors can be learned through social encounters. In particular, the model claims that aggressive behaviors will arise through an interaction between personal (relatively stable long-term factors and processes—e.g., sexist attitudes or trait aggression) and situational (more temporary short-term proximal risk factors and processes—e.g., exposure to sexualized video game content) variables. As shown in Figure 1, these two systems reciprocally influence each other and the interaction between personal and situational variables will influence the present internal state through three interconnected routes that include aggressive thoughts, angry feelings, and physiological arousal. This process will generate an immediate appraisal of the situation. For instance, confronted with a testimony of rape, this can result in rape myth acceptance, i.e., holding the victim, and not the perpetrator, partially or fully responsible for being raped. If the appraisal is judged to be unsatisfactory and if the person has sufficient time and/or cognitive resources, the situation might be reappraised and may lead to a thoughtful action (e.g., revising the initial judgment). However, if the person judges the immediate appraisal to be satisfactory (e.g., it aligns with the person’s sexist attitudes) and/or if the person does not have sufficient time and/or cognitive resources available at the moment (e.g., due to performing a cognitively demanding activity such as a video game), s/he will not reappraise the situation and this may lead to an impulsive behavior. By extending the GAM to the context of sexualization in video games, this exposure is therefore considered as being a situational variable that may influence a person’s current internal state and, as a result, influence his/her behavior. Based on this model, we focused on the impact of sexualized video game content on rape myth acceptance, and on the mediating effect of humanness.

Degree of humanness accorded to the victim and the perpetrator was chosen because, as mentioned previously, in most video games, female characters are sexualized and objectified, which leads to dehumanization (Burgess et al., 2007; Vaes et al., 2011; Loughnan et al., 2013; Puvia and Vaes, 2013; Summers and Miller, 2014; Blake et al., 2016). Dehumanization occurs when a person is treated as an animal, an object, or—in a more subtle way—as not completely human (Haslam, 2006; Gervais et al., 2013). Two main forms of humanness can be denied of the person: human uniqueness and human nature (Haslam, 2006). Human uniqueness corresponds to attributes that are seen as distinguishing humans from other animals and reflects social learning and refinement. Its denial is called “animalistic dehumanization” and refers to the fact that the person is considered more animal than human. Human nature corresponds to features that are fundamental and shared by all humans, such as emotionality, agency, warmth, and cognitive flexibility (Haslam, 2006). Its denial is termed “mechanistic dehumanization,” meaning that the person is considered to be an object or an automaton. Humanness has already been related to RMA (i.e., victim blame) in various studies. One study showed that attitudes toward rape victims are predicted by the implicit association between women and animals (Rudman and Mescher, 2012). Another study showed that when women are denied of their specific human qualities, they are considered as more likely to become targets of sexual aggression (Blake et al., 2016). These studies let us assume that RMA is at least partially explained by the degree of humanness granted to women. Given that sexualization and objectification lead to dehumanization, a measure of the humanness level granted to both the victim and the perpetrator was included as a mediator of the link between sexualized video game content and RMA.

Another important variable to take into account is the availability of cognitive resources. According to the GAM, the interaction of personal and situational variables leads to the immediate appraisal of a situation (e.g., a testimony of rape) and therefore to the most spontaneous and the most accessibly linked thoughts and feelings. As presented previously, concerning a testimony of rape, these thoughts can lead to the dehumanization of the victim and to the adherence to RMA. The situation can be reappraised, but in order to do this, the person needs sufficient cognitive resources. The expenditure of cognitive resources is an inherent feature of video games. Playing video games requires more cognitive resources than other types of media because the player needs to concentrate on, and interact with, the media (Lin, 2013). For this reason, we suppose that cognitive resources consumed by a video game interact with the appraisal decision processes and lead to an attitude consistent with our stereotypes about rape. Therefore, we posit that the quantity of available cognitive resources can moderate the relation between sexualization and both humanness and the responsibility accorded to the victim/perpetrator (state rape myth acceptance). Only one study (Read et al., 2018) has examined the potential moderating effect of cognitive load between sexualization and RMA. In this study, participants were exposed to sexualized or non-sexualized video game characters. Further, cognitive load was manipulated by asking participants to remember either two or seven symbols. Results showed that being exposed to a video game with sexualized content, in addition to having few available cognitive resources, caused a diminution of RMA. However, this study used a trait measure of RMA (i.e., this measure was designed to evaluate a stable, dispositional aspect of the person) and not a state measure of RMA (i.e., measure aiming to evaluate a reaction to a situation). Thus, it is unclear whether their results are due to their manipulation of sexualized content (thus influencing participants’ responses to the RMA scale), or due to a sample bias (the selected participants may not be representative of the population). Further, cognitive load was manipulated by asking participants to memorize and recall symbols during gameplay. However, gameplay itself can influence cognitive load and therefore, even participants in the low cognitive load condition might have lacked sufficient available cognitive resources when trying to reappraise the situation.

We also included several covariates. Based on the GAM, we know that aggressive behaviors will arise, not only from the effect of situational variables, but from their interaction with personal variables. For this reason, it seems obvious to include as covariates not only a measure of aggressive personality as proposed by the GAM, but also a measure of benevolent sexism. Individuals who score high on benevolent sexism strongly endorse the belief that women are pure and should be protected. However, such a belief implies that women must behave in ways that allow them to be protectable (Glick et al., 1997). If women are perceived as violating benevolent sexist expectations, individuals scoring high on benevolent sexism may perceive them as no longer deserving protection (Glick et al., 1997). This is in line with study results showing that individuals high on benevolent sexism are more likely to react negatively to rape victims who can be viewed as violating social norms concerning appropriate conduct for women (e.g., Viki and Abrams, 2002), an idea reflected in some rape myth beliefs and, thus, in the scenario we used for the present study: the female character was drunk, flirting with the male character, and described as provocatively dressed. Finally, we asked participants if they identify themselves as a “gamer,” because video game experience most likely impacts the necessary cognitive load during the game.

In view of the above facts, the objective of the present study is to examine: (1) the impact of sexualized content of video games on state RMA, (2) the potential moderating effect of cognitive load, and (3) the mediating effect of humanness. Measures of trait aggression, benevolent sexism and gamer identification where included as covariates. These issues will be examined while at the same time addressing the limitations of previous studies and introducing new innovations. In fact, the mediating effect of humanness (of both the victim and the perpetrator) concerning the relation between sexualized content of video games and RMA will be examined, and this to the best of our knowledge for the first time in the context of a study on sexualized video games. Furthermore, studies examining the effects of sexualized video game content on RMA have resulted in mixed results, probably due to some limitations concerning the methodology. One study showed that sexualized content of video games can directly increase RMA (Driesmans et al., 2015), and another study showed that video games indirectly increased RMA through increases of self-objectification (Fox and Potocki, 2016). Finally, two studies showed no effect of sexualized video game content on RMA (Dill et al., 2008; Beck et al., 2012). However, as previously emphasized, these studies have two main limitations. First, they all used a trait measure of RMA. Second, several studies possess poor ecological validity. For example, in one study (Beck et al., 2012), participants watched another person play the video game instead of having participants play the video games themselves. Therefore, the present study used a state (and not trait) rape myth acceptance measure. Specifically, participants read a rape date story and judged the victim’s and the perpetrator’s degree of responsibility and degree of humanness. In addition, in order to create a situation as ecological as possible, participants were asked to play a video game. Cognitive load was manipulated by modifying the level of difficulty of the game. A pretest showed that this manipulation of cognitive load was effective.

To conclude, our hypotheses were: (1a) when exposed to a sexualized video game, participants will score high on the state RMA measure and thus will hold the victim (and not the perpetrator) partially or fully responsible for being raped (rape victim blame), especially in conditions of high cognitive load; (1b) degree of humanness will mediate the relation between sexualization and rape victim blame: the sexualized condition will decrease the human uniqueness score and the human nature score attributed to the victim, which will increase rape victim blame; (2a) when exposed to a sexualized video game, rape perpetrator blame will diminish, especially in conditions of high cognitive load; and (2b) degree of humanness will mediate the relation between sexualization and rape perpetrator blame: the sexualized condition will increase the human uniqueness score and the human nature score attributed to the perpetrator, which will diminish perpetrator blame.

## Materials and Methods

### Participants

Participants were 142 university students (71 male and 71 female) aged between 18 and 27 years (*M* = 21.66, *SD* = 1.53) recruited from a Belgian university, through social networks and by word of mouth. Participation was voluntary and unpaid. Among the 142 participants, 65 identified themselves as video game players. They reported spending between 1 and 20 h per week playing video games (*M* = 7.92, *SD* = 5.24).

### Materials

#### Video Games

All participants played the same video game (Ultra Street Fighter IV). In this fighting game, the player embodies a character who fights with punches and kicks against another (single) opponent who counterattacks. Different keys on the computer keyboard allow participants to perform punches, kicks, jumps, crouches and to combine different moves. The goal is to exhaust the opponent’s health meter or have more health than the opponent when the time runs out. This game was chosen as it offers the possibility to select female characters, but also because it offers the possibility to choose between very covering clothes and very exposing clothes (the characters being almost naked). Equally important, this game makes it easy to decide the level of difficulty and allows participants to play without having to familiarize themselves with complicated rules (the only required knowledge is how the keyboard keys are related to various movements). Two variables were manipulated in this study: sexualization and cognitive load. Sexualization was manipulated by changing the outfit of the two female characters (i.e., the character manipulated by the participant and his opponent). In the highly sexualized condition, both characters wore a revealing swimsuit (the characters were completely naked except for the breasts and gender), whereas in the non-sexualized condition, both characters were fully clothed (only the face and hands were visible). Cognitive load was manipulated by modifying the action of the computerized opponent. In the low cognitive load condition, the opponent was programmed to never fight back, whereas in the high cognitive load condition, the opponent was programmed to fight back. In both conditions, participants were asked to learn the action produced by each button and their combination.

#### Cognitive Load Pretest

Pre-test participants (who were not included in the main study) were recruited via social networks. Both cognitive load conditions were pre-tested using a dual-task methodology (Paas et al., 1994; Marcus et al., 1996; Brünken et al., 2002). We used an objective and a subjective method to measure the cognitive load manipulation, respectively, an auditory 2-back task that the participant had to perform while playing the video game, and a mental effort scale (Haji et al., 2015) presented after playing the video game. For the auditory 2-back task, participants were asked to listen to an audio recording of 300 numbers that were presented with an interval. Participants were required to use a verbal signal when the number heard (i.e., the target) was the same as the second to last number they heard. In this 2-back task, 20% of the numbers were a target. Omissions and false alarms were recorded. Participants were instructed to primarily focus on the video game while at the same time to also pay attention to the auditory 2-back task. The mental-effort scale (Haji et al., 2015) consisted of a single item ranging from 1 (*very low mental effort*) to 9 (*very high mental effort*). When answering, participants were asked to only consider the mental effort provoked by the video game and not to consider the mental effort provoked by the secondary task.

Nineteen participants were randomly assigned to either the high cognitive load condition (*N* = 10) or the low cognitive load condition (*N* = 9). As expected, participants in the high cognitive load condition, compared to participants in low cognitive load condition, committed significantly more omissions during the verbal 2-back task [*t*(17) = −2.49; *p* < 0.05, *d* = 1.21] and rated the mental effort provoked by the video game as higher [*t*(7) = −2.20; *p* < 0.05, *d* = 1.07].

#### Date Rape Judgment Task

The date rape task consisted of a written scenario describing a college party. The scenario involves a male and a female character that are meeting for the first time and flirting together. At some point in the story, the male character became more sexually insistent, while the female character’s interest decreases progressively. The story ends up with the male character having sex with the female character albeit without her consent (i.e., raping her). Originally this scenario was separated into two parts (Hull et al., 2016), one in which the story is told from the female character’s point of view and one from the male character’s point of view. We made two modifications to this scenario. First, the two parts from the story were combined in a single version, bringing together the male character’s point of view and the female character’s point of view. Second, we renamed the characters with more common Belgian names (i.e., “Sophie” and “Arnaud”). The scenario involved several elements that evoke rape myths: both characters were drunk, the female character was flirting with the male character, and was described as being provocatively dressed, and the male character showed a “moderate” form of violence (grabbing, pushing and restraining). After reading the scenario, participants answered different questions concerning the degree of responsibility of the female and the male characters. The full scenario of the date rape judgment task can be found in the Supplementary Material.

### Questionnaires

#### Demographic Information and Video Game Consumption

Participants reported their gender and age. They also reported the average number of hours they spend playing video games each week, and their degree of familiarity with the video game used in the study (estimate of the total number of hours spent on this game). Familiarity has been added as a covariate.

#### Gamer Identification

Identification was measured via a set of 4 questions: “I see myself as a gamer,” “I like being a gamer,” “I feel a strong connection with other gamers” and “I identify with other gamers.” Participants rated the statements on a scale ranging from 1 = S*trongly agree* to 5 = *Strongly disagree*. Internal consistency was excellent (α = 0.91).

#### Victim and Perpetrator Blame

Victim and perpetrator blame were assessed using four items issued from a previous study on the same topic (Bernard et al., 2015). The items were “How much do you think Sophie/Arnaud should blame herself/himself for what happened?,” “How much control do you think Sophie/Arnaud had over the situation?,” “Do you think this incident could have been avoided by Sophie/Arnaud?,” and “How much do you think that Sophie/Arnaud is responsible for the way things turned out?” Items were answered using a 7-point response scale ranging from 1 = *Not at all* to 7 = *Completely or totally*. These measures, representing the dependent variable, were used as state measures of rape myth acceptance. A high score on this measure for the assessment of the female character indicates a strong acceptance of rape myth, while a high score on the male character assessment indicates a weak acceptance of rape myth. Internal consistency in this study was good for both victim and perpetrator blames (Cronbach α = 0.83 and 0.75, respectively).

#### Humanness Scale

To assess humanness of the victim and of the perpetrator, participants were given a list of 20 traits, taken from Bastian and Haslam (2010), that included five positive human uniqueness traits (broadminded, conscientious, humble, polite, thorough—French translation: ouvert d’esprit, consciencieux, humble, poli, rigoureux), five negative human uniqueness traits (disorganized, hard-hearted, ignorant, rude, stingy—French translation: désorganisé, insensible, ignorant, grossier, avare), as well as five positive human nature traits (active, curious, friendly, helpful, fun-loving—French translation: actif, curieux, amical, serviable, bon vivant) and five negative human nature traits (impatient, impulsive, jealous, nervous, shy—French translation: impatient, impulsif, jaloux, nerveux, timide). Participants were asked if these traits could be attributed to the victim and the perpetrator using a 7-point Likert scale ranging from 1 = *Strongly disagree* to 7 = *Strongly agree.* The humanness score was therefore composed of two distinct scores: human uniqueness (the average of positive and negative human uniqueness scores) and human nature (the average of positive and negative human nature scores).

#### Trait Aggression

Finally, participants completed a French version (Genoud and Zimmermann, 2009) of the Aggression Questionnaire (AQ; Bryant and Smith, 2001), which contains 12 items (e.g., “I have threatened people I know”) that are scored using a 6-point response scale ranging from 1 = *Not at all like me* to 6 = *Completely like me* (Cronbach α = 0.71).

#### Benevolent Sexism

Participants also completed the 11-item benevolent sexism subscale (Dardenne et al., 2006) of the Ambivalent Sexism Inventory (ASI; Glick and Fiske, 1996). All items are scored using a 5-point Likert scale ranging from 0 = Totally disagree to 5 = Totally agree. Internal consistency is good (Cronbach α = 0.84).

#### Rape Myth Acceptance

The Updated Illinois Rape Myth Acceptance Scale (Mcmahon and Farmer, 2011) was used as a trait measure of rape myth acceptance, and was included as a covariate in the analyses. The scale was translated and back translated to create a French version for the purpose of this study. This scale contains 22 items (e.g., “If a girl is raped while she is drunk, she is at least somewhat responsible for letting things get out of hand.”) that are scored using a 5-point Likert scale ranging from 1 = *Strongly Agree* to 5 = *Strongly Disagree*. The French version possesses good levels of internal consistency (α = 0.89).

### Procedure

Upon arrival, participants gave their informed written consent and were told that they would participate in a study about the impact of various types of media on the judgments about agentivity (capacity of action). Participants were told that they would be exposed to stories through various types of media and that they would thereafter be asked to judge the degree of agentivity of each protagonist in the story. First, they completed questions about demographics and their identification as a gamer. Based on the procedure used by Read et al. (2018), participants were first subjected to the experimental condition, and then, directly afterward, completed the different questionnaires. So, after completing demographic questions, participants were told that they could potentially be exposed to 10 types of media, but that would make the experiment too long. Therefore, participants had to choose two numbers between 1 and 10. In reality, participants were assigned the video game and the rape date story, no matter which number they chose. Immediately after, they were led to a computer with a 24” screen and randomly assigned to one of the two sexualization conditions (i.e., sexualized vs. non-sexualized) and one of the two cognitive load conditions (i.e., high cognitive load vs. low cognitive load). Participants played the video game for 15 min using an Xbox controller for computer. Few studies have examined the minimum time required to observe the effects of video game playing on people’s attitudes or behaviors. Based on a study looking at increasing surgical skills through computer simulation, it seems that playing for an accumulated time exceeding 30 min is sufficient to improve participants skills and to consider them as experienced “gamers” (Hsu et al., 2004). In our study, and in the high cognitive load condition, it was necessary to avoid that participants achieved a level of “expertise” for the video game in order to ensure that the impact of cognitive load was high in this condition (this is not applicable for the low cognitive load condition since, in this condition, the opponent does not defend her/himself and therefore does not require any cognitive resources anyway.) For this reason, we made sure that the playing time was below 30 min. A playing time of 15 min was therefore decided, as this corresponds to the most used playing time in studies on video games (e.g., Driesmans et al., 2015). Immediately after playing, participants read the rape date story and answered the questions about victim and perpetrator blame, and about victim and perpetrator humanness. The order of the measure of the dependent variable and the mediator was fixed.

After that, participants completed the Aggression Questionnaire, the Benevolent Sexism scale and the Illinois Rape Myth Acceptance Scale, and in that order. A debriefing followed, which included a check for suspicion. Each participant who guessed the real purpose of the study was removed from the sample. Based on this criterion, 58 participants (29%) were removed from the initial sample (*N* = 200), resulting in a final sample of 142 participants.

## Results

**FIGURE 2:**
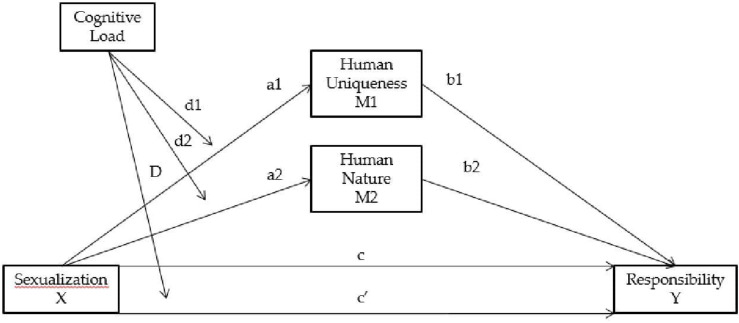
The proposed model whereby the relation between sexualization and responsibility is mediated by degree of Humanness (Human Uniqueness and Human Nature) and the relations between sexualization and responsibility and between sexualization and degree of Humanness are moderated by Cognitive Load.

Regression analysis was used to test the hypotheses. Using model 8 from the PROCESS macro for SPSS (Hayes, 2015), two models were computed (Figure 2), one for the victim and one for the perpetrator. In the victim model, victim blame was the dependent variable (used as proxy for state RMA), sexualization was the independent variable, humanness of the victim was the mediator, and cognitive load was the moderator. In the perpetrator model, perpetrator blame was the dependent variable (used as proxy for state RMA), sexualization was the independent variable, humanness of the perpetrator was the mediator, and cognitive load was the moderator. In both models, Sexualization was coded −1 for low and 1 for high. Similarly, cognitive load was coded −1 for low and 1 for high. Trait rape myth acceptance, gamer identification, benevolent sexism, familiarity with the game, and trait aggression were included as covariates for both models. All variables were standardized.

In the first model, cognitive load was examined as a moderator of the relation between sexualization and victim blame, and as a moderator of the relation between sexualization and degree of humanness attributed to the victim (Table 1). The only statistically significant moderation was between sexualization and responsibility of the victim. The slope in the low cognitive load condition was not statistically significant, *b* = −0.042, *t* = −0.423, *SE* = 0.099, *p* = 0.673, whereas the slope in the high cognitive load condition was statistically significant, *b* = 0.274, *t* = 2.737, *SE* = 0.100, *p* = 0.007. Further results showed that victim blame was significantly predicted by familiarity with the game (*b* = 0.234, *t* = 3.17, *SE* = 0.074, *p* = 0.002) and trait rape myth acceptance (*b* = −0.539, *t* = −6.90, *SE* = 0.078, *p* < 0.001). Human uniqueness was predicted by gamer identification (*b* = −0.113, *t* = −2.06, *SE* = 0.055, *p* = 0.042) and familiarity with the game (*b* = 0.153, *t* = 2.85, *SE* = 0.054, *p* = 0.005). Human nature was predicted by benevolent sexism (*b* = −0.139, *t* = −2.03, *SE* = 0.068, *p* = 0.044). Further, the first model suggested a relation between sexualization and victim blame that is mediated by the degree of humanness of the victim. None of the indirect paths were significant.

**TABLE 1:** Path coefficients, indirect effects and 95% bias-corrected Confidence Intervals for multiple mediation analysis (bootstrap of 20,000 resamples) and moderation analysis.

	***R*^2^**	***F***	***p***	**Path**	***b***	***SE***	***t***	***p***	**95% CI**	
									**Lower**	**Upper**
Victim blame (Y)	0.391	8.41	<0.001							
				Direct effect Sexualization (c’)	0.116	0.070	1.66	0.099		
				Cognitive load (D)	0.002	0.069	0.02	0.983		
				Interaction (c’ X D)	0.158	0.071	2.23	0.027*		
				HU (b1)	−0.150	0.128	−1.17	0.242		
				HN (b2)	0.146	0.109	1.34	0.182		
				RMA	−0.539	0.078	−6.90	<0.001*		
				GI	0.143	0.074	1.93	0.056		
				GA	0.019	0.074	0.26	0.792		
				BS	−0.009	0.080	−0.11	0.910		
				FAM	0.234	0.074	3.19	0.002*		
SHU (M1)	0.154	3.03	0.004							
				Sexualization (a1)	0.055	0.053	1.04	0.301		
				Cognitive load (d1)	−0.034	0.052	−0.65	0.516		
				Interaction (a1 X d1)	−0.068	0.053	−1.30	0.197		
				RMA	−0.106	0.058	−1.83	0.069		
				GI	−0.113	0.055	−2.06	0.042*		
				GA	−0.089	0.055	−1.61	0.109		
				BS	0.043	0.059	0.74	0.463		
				FAM	0.153	0.054	2.85	0.005*		
SHN (M2)	0.095	1.75	0.092							
				Sexualization (a2)	0.075	0.061	1.22	0.225		
				Cognitive load (d2)	−0.009	0.061	−0.15	0.885		
				Interaction (a2 X d2)	0.028	0.062	0.45	0.650		
				RMA	−0.134	0.068	−1.97	0.050*		
				GI	−0.103	0.065	−1.60	0.112		
				GA	−0.064	0.064	−0.10	0.320		
				BS	−0.139	0.068	−2.03	0.044*		
				FAM	−0.122	0.063	1.94	0.055		
Indirect effects										
				a1b1		0.028			−0.018	0.093
				a2b2		0.022			−0.035	0.060

Effects of sexualized video game content on victim blame through degree of humanness attributed to the victim (Human Uniqueness and Human Nature) and the effect of cognitive load as a moderator between these variables. Interaction, Interaction between Sexualization and Cognitive Load; HU, Human Uniqueness; HN, Human Nature; RMA, Rape Myth Acceptance; GI, Gamer Identification; GA, General Aggression; BS, Benevolent Sexism; FAM, Familiarity with the game. *Significant result.

In the second model, cognitive load was examined as a moderator of the relation between sexualization and perpetrator blame, and as a moderator of the relation between sexualization and degree of humanness attributed to the perpetrator (Table 2). None of the interactions were significant. Further results showed that perpetrator blame was significantly predicted by general aggression (*b* = 0.187, *t* = 2.20, *SE* = 0.085, *p* = 0.029) and trait rape myth acceptance (*b* = 0.370, *t* = 4.21, *SE* = 0.088, *p* < 0.001).

**TABLE 2:** Path coefficients, indirect effects and 95% bias-corrected Confidence Intervals for multiple mediation analysis (bootstrap of 20,000 resamples) and moderation analysis.

	***R*^2^**	***F***	***p***	**Path**	***b***	***SE***	***t***	***p***	**95% CI**	
									**Lower**	**Upper**
Perpetrator blame (Y)	0.199	3.26	0.001							
				Direct effect Sexualization (c’)	0.043	0.081	0.53	0.600		
				Cognitive load (D)	0.010	0.080	0.13	0.898		
				Interaction (c’ X D)	−0.073	0.082	−0.89	0.375		
				HU (b1)	0.017	0.132	0.13	0.899		
				HN (b2)	0.208	0.112	1.86	0.065		
				RMA	0.370	0.088	4.21	<0.001*		
				GI	−0.091	0.084	−1.08	0.280		
				GA	0.187	0.085	2.20	0.029*		
				BS	−0.057	0.089	−0.64	0.522		
				FAM	−0.012	0.082	−0.148	0.882		
HU (M1)	0.074	1.34	0.232							
				Sexualization (a1)	0.090	0.054	1.66	0.100		
				Cognitive load (d1)	0.019	0.054	0.35	0.725		
				Interaction (a1 X d1)	−0.089	0.054	−1.63	0.105		
				RMA	−0.004	0.060	−0.06	0.952		
				GI	−0.073	0.057	−1.28	0.203		
				GA	0.112	0.057	1.98	0.050		
				BS	−0.027	0.060	−0.44	0.659		
				FAM	−0.029	0.055	−0.520	0.604		
HN (M2)	0.044	0.76	0.639							
				Sexualization (a2)	−0.045	0.064	−0.71	0.482		
				Cognitive load (d2)	−0.058	0.064	−0.91	0.365		
				Interaction (a2 X d2)	0.068	0.064	1.06	0.290		
				RMA	−0.025	0.070	−0.35	0.727		
				GI	−0.017	0.067	−0.26	0.797		
				GA	−0.014	0.067	−0.21	0.834		
				BS	−0.086	0.071	−1.21	0.227		
				FAM	0.086	0.065	1.31	0.191		
Indirect effects										
				a1b1		−0.003			−0.059	0.047
				a2b2		0.028			−0.020	0.047

Effects of sexualized video game content on perpetrator blame through degree of humanness attributed to the perpetrator (Human Uniqueness and Human Nature), and the effect of cognitive load as a moderator between these variables. Interaction, Interaction between Sexualization and Cognitive Load; HU, Human Uniqueness; HN, Human Nature; RMA, Rape Myth Acceptance; GI, Gamer Identification; GA, General Aggression; BS, Benevolent Sexism; FAM, Familiarity with the game. *Significant result.

## Discussion

This study examined the impact of sexualized video game content on rape victim and on perpetrator blame. Only one other study has previously examined the moderating effect of cognitive load (Read et al., 2018). In addition to this, it is the first study to examine the mediating effect of humanness in the context of a video game.

Consistent with the first hypothesis (1a), victim blame was significantly more likely to occur when the participant was exposed to a sexualized video game, but only in conditions of high cognitive load. The second hypothesis (1b) was not supported. The impact of sexualized video game content on victim blame was not mediated by humanness. Further, the third hypothesis (2a) and the fourth hypothesis (2b) were not supported. Results did not show a direct effect of sexualized content on perpetrator blame and cognitive load did not moderate this relation. Moreover, the impact on sexualized video game content on perpetrator blame was not mediated by humanness.

Cognitive load seems to be an important variable to account for as it appears to moderate the impact of exposure to sexualized video game content on victim blame. We could only find one previous study that measured cognitive load when examining the impact of sexualized video game content on RMA (Read et al., 2018) and results from this study went in the opposite direction as in the present study. Certain differences between these studies may explain this, such as the fact that the previous study used a trait measure of rape myth acceptance and did not separate victim and perpetrator blame. When we look at the methodologies of other studies that have tested the impact of sexualized video game content on RMA (albeit without controlling for high vs. low cognitive load), we remain convinced that cognitive load is the key to understanding the inconsistent results in these studies. Indeed, when a study used a manipulation that is susceptible to cause high cognitive load (e.g., by asking participants to actually play a video game; Driesmans et al., 2015), results showed an impact of sexualized video game content on RMA. In contrast, other studies (Dill et al., 2008; Beck et al., 2012) used conditions that are not susceptible to cause high cognitive load (e.g., showing images from video games or asking participants to watch another person play the video game), and in those studies, sexualized video game content did not influence RMA. The present study used both conditions (a low cognitive load condition and a high cognitive load condition) and showed results that are consistent with both types of studies (i.e., an effect of sexualized game content on RMA in the high cognitive load condition and an absence of an effect in the low cognitive load condition). These results are in line with the GAM (Bushman, 2017; Anderson and Bushman, 2018), which posits that impulsive aggressive attitudes, which emerge from the immediate appraisal of a situation, only take place if we are not able to reappraise the situation (e.g., if we do not have enough available cognitive resources). These results are also in line with the well-known phenomenon called “stereotype inhibition,” which proposes that people inhibit the stereotypes they hold in order to avoid social disapproval. As mentioned previously, RMA is based on stereotypes surrounding sexual violence (Buddie and Miller, 2001). These stereotypes are socially undesirable, so people inhibit them. However, if detecting the presence of stereotypic ideas is relatively easy (Macrae and Bodenhausen, 2000), inhibiting these stereotypes requires increased attentional resources and can only occur when these cognitive resources are available (Wegner, 1994).

In addition, the present study is the first to examine the impact of sexualized video game content on perpetrator blame. The results did not confirm our third hypothesis. Indeed, neither a direct impact of sexualized game content nor a moderated effect by cognitive load was found on perpetrator blame. To the best of our knowledge, only one study has previously examined the impact of sexual objectification on perpetrator blame (Bernard et al., 2015). This study showed that exposure to sexual objectification (using a picture) decreases the responsibility of the perpetrator in a stranger rape context. The difference in results between that study and the present study might be due to the context of the rape. In Bernard et al. (2015), participants were asked to read a newspaper article in which sexual objectification was manipulated. In this newspaper, little information is given about the victim (she is a famous young model coming back from a banquet in honor of the official launch of a new brand of lingerie and she tells reporters that she will return to photo shoots as soon as possible) and, more importantly, on how she perceives the situation and her behaviors to get out of it (participants are only told that “her attempts to struggle were futile”). Even less information is given about the perpetrator (participants only know his name, his age and that he has no serious alibi). The present study used a rape date story that provided information about the perpetrator (i.e., his personality, his attitude, his perception of the situation), but also about the victim (i.e., her personality, her attitude, her perception of the situation and her attempts to get out of the situation). In the context of the rape date story used in the present study, the perpetrator and the victim are both personalized. In a different study (Bernard et al., 2016), more personalized details were provided about the perpetrator and the victim of sexual harassment and results showed, as in the present study, that there was no significant effect of exposure to sexualized media on perpetrator blame. In this study, a scenario is presented where Anne, a second-year university student, takes part in weekly tutoring sessions to improve her English skills. She is tutored by John, a senior graduating with honors in English. In this scenario, participants are informed that when John, at each tutoring session, tells Anne how much he finds her attractive, she “always changed the subject, getting them back on the topic they were supposed to be discussing.” This clearly demonstrates Anne’s discomfort and her systematic attempts to change the subject and get out of the situation. It is therefore possible that giving information about the victim (her perception of the situation and her attempts to stop it) may have little or no effect on the responsibility given to her, but may still prevent the perpetrator’s responsibility from diminishing significantly (as it is the case when no information is given about the victim’s behavior and perception). It should also be pointed out that most participants in psychology studies are students and are therefore more likely to compare themselves and feel concerned by scenarios that take place in the academic world, and that most students will find it highly difficult to relate to the fashion and modeling world. Feeling that they belong to the same group as the victim most likely leads participants to not undervaluing the offender’s responsibility in a significant way.

We also assumed that the impact of sexualization on victim/perpetrator blame was mediated by the degree of humanness accorded to the victim/perpetrator (second and fourth hypotheses). These hypotheses were not supported. Regarding the second hypothesis, humanness did not mediate the impact of sexualized content on victim blame. Moreover, no direct effect of sexualization on humanness was observed. These results are not in line with previous studies on the dehumanization of sexualized women. In these studies (Vaes et al., 2011; Loughnan et al., 2013; Puvia and Vaes, 2013; Blake et al., 2016), when a woman was sexualized, she was also dehumanized. One possible explanation lies in the differences in methodology. In Loughnan et al. (2013), participants were asked to assess the degree of humanity of sexualized (or non-sexualized) women based on a picture and a description before receiving information about rape. In other studies (Vaes et al., 2011; Puvia and Vaes, 2013), implicit associations are used, indicating an implicit tendency to dehumanize the objectified sexualized women. In the present study, a lot of information is given about the context of the rape and about the victim herself, before assessing her degree of humanness. Participants have the information of the rape when assessing the degree of humanness of the victim and have time to think about all the information they have about the situation and the female character of the rape judgment task. This may partly explain these results. The more personal information we have about a person, the more we can identify with this person, develop empathy and find it difficult to disregard all the characteristics that make her a human being. This explanation is in line with the results from a study examining the role of dehumanization in attitudes toward the social exclusion and rehabilitation of sex offenders (Viki et al., 2012). In this study, it was found that good quality contact between correctional staff and sex offenders was related to less dehumanization and more support for rehabilitation (Viki et al., 2012). Good quality contact may have led the correctional staff to have more personal information about the sex offenders, possibly resulting in more identification with the criminal or the development of empathy. Thus, if sexualization leads to a dehumanization of the victim, it is possible that this effect no longer exists once we feel close to the victim and perhaps develop empathy. However, as a result of this reasoning, a significant increase in the degree of humanity accorded to the aggressor (as posed by the fourth hypothesis) is expected. This is not supported by our results. One possible explanation lies in the current societal context. Indeed, contrary to the other studies cited, ours takes place after 2017, that is, after the Weinstein affair, which perhaps contributes to the fact that it is no longer socially accepted to “humanize” a rapist, and that this effect is therefore no longer significant.

It would be important to investigate how one feels about the responsibility attributed to the victim and the abuser, given that this can be perceived very differently. As explained by the GAM, if an individual has the necessary cognitive resources s/he will be able to re-evaluate a situation and thus go against her/his first stereotyped thoughts and feelings. While it is true that regular video game players increase their expertise and thus could free up cognitive resources that can allow them to adequately assess the situation, we also know that the difficulty of video games increases with each level. As is often the case, the solution will probably lie more in the representation of female characters, which can be portrayed in a less sexualized manner, rather than in the game itself and the related degree of cognitive load.

### Theoretical Significance

In general, the results are consistent with the General Aggression Model (GAM, Bushman, 2017; Anderson and Bushman, 2018). The situational variable of sexualized game content increased negative attitudes toward female rape victims, but only in the high cognitive load condition. Thus, cognitive load moderated the impact of sexualized game content on negative attitudes. In other words, cognitive resources consumed by a video game interact with the appraisal decision processes leading to a more impulsive and negative judgment of a rape victim. However, the exact temporality of this relation remains unclear. In this study, the video game directly caused cognitive load. Whether cognitive load directly influences the appraisal of the victim’s responsibility, or only impacts the decision to express that opinion, is unclear. Future studies should try to disentangle that temporality (e.g., by introducing cognitive load after exposure to a video game with sexualized content).

One main contribution of these results is that it confirms the particular role of cognitive load caused by video games. In this study, cognitive load was directly caused by features from the video game (i.e., degree of difficulty of the video game). However, it remains unclear which particular characteristics of the video game lead to high cognitive load. For example, future studies could test if high cognitive load results from features such as interactivity (e.g., the number of buttons needed to play the game), reactivity (e.g., the number of actions required by the video game), or the degree of immersion in the video game (e.g., by using virtual reality). Further, it would be important to manipulate some of these characteristics in other types of media (e.g., film and television) to test if the impact is similar to video games. Also, it would be interesting to include, in addition to the subjective gamer identification measure, an objective measure of the degree of experience of the video game player. If high cognitive load is only determined by the difficulty (level) of the game itself, and not by features such as interactivity, reactivity, or degree of immersion, then experienced and novice players might be affected in different ways. Thus, future studies should focus on this variable and recruit enough video game players to allow a comparison between groups of novice, intermediate, and experienced players, while taking into account the type of games usually played by each participant.

### Limitations

The present study has some limitations. The video game that was chosen for the present study was not ideal. Since we wanted to have as high a level of ecological validity as possible, we wished to include a video game that people actually use. In addition, we needed to find a video game that included female characters and that enables a manipulation of both degree of cognitive effort and degree of sexualization. The only video game at the time of the study that answered to all these criteria was Ultra Street Fighter IV. However, cognitive load may have been confounded with aggression. Indeed, in the high cognitive load condition, the opposing character defends himself, which is not the case in the low cognitive load condition. Furthermore, degree of sexualization was manipulated by modifying outfits of the female video game characters. Given that Ultra Street Fighter IV is a fighting video game, the female characters possess features that are stereotypically inconsistent with the usual submissive role of female characters in video games. For instance, it was easy to see the strong muscles of the sexualized characters. Such stereotype-inconsistent information might also act as a confounding variable and might prime other concepts such as agentivity, which is known to mediate the relation between sexualized game content and risk of sexual aggression (Blake et al., 2016). This agentivity of the female sexualized character might also explain why our manipulation did not prime dehumanization. Future studies should thus find a video game that addresses these potential limitations, that is, a video game that not only enables a manipulation of degree of cognitive load albeit without manipulating aggression, but also that does not include potentially stereotyped-inconsistent information (or try to determine their specific impact by using a different type of video game). In addition to the aforementioned limitations, most of the participants never played video games and only 65 (less than half of the overall sample) identified themselves as gamers. Among the gamers, the number of hours spent gaming per week varied from 1 to 20, with an average of 8 h per week. The degree of ease of playing video games, thus, varied quite considerably within the sample. Given the importance of cognitive load, future studies should ensure that this aspect has been more optimally controlled.

## Conclusion

In conclusion, the present results contribute to a better understanding of the impact of sexualized video game characters on rape victim blame. Our results show that playing a video game containing sexualized female characters increases rape victim blame, when participants’ cognitive resources are low. Negative attitudes toward women in general, and rape myth attitudes in particular, are an important issue in our society and one of its underlying causes might be the sexualized content of video games.

## Data Availability Statement

The datasets presented in this study can be found in online repositories. The names of the repository/repositories and accession number(s) can be found below: https://doi.org/10.18710/FDJEIB.

## Ethics Statement

The study involving human participants were reviewed and approved by the Comité Éthique de la FPLS, Université de Liège, Belgique. The participants provided their written informed consent to participate in this study.

## Author Contributions

TN, FL, and JB contributed to the design and implementation of the research, to the analysis of the results, and to the writing of the manuscript. All authors contributed to the article and approved the submitted version.

## Conflict of Interest

The authors declare that the research was conducted in the absence of any commercial or financial relationships that could be construed as a potential conflict of interest.
