# Supplementary material for: The Impact of Sexualized Video Game Content and Cognitive Load on State Rape Myth Acceptance
Source: Front Psychol. 2021 Mar 15;12:614502. doi: 10.3389/fpsyg.2021.614502 (PMC8005642; doi:10.3389/fpsyg.2021.614502)
Supplement: Supplementary file 1 [file Data_Sheet_1.PDF]

RAPE DATE STORY

One day, Sophie and Arnaud enjoy alcoholic drinks separately with their friends, when they receive a text message. The SMS invites them to a “CEO and sexy secretary” costume party that same evening. Arnaud dresses up with a nice pair of pants, a shirt and a tie. Meanwhile, Sophie puts on a tight straight skirt and a button-down blouse that reveals her bra.

Arnaud arrives at the party first and is led straight into the room which contains several beer crates. Sophie arrives in the same room a little later. After a drink or two, they both join the group in the room next door to play drinking games. While watching the other players, they end up noticing each other. They find each other quite cute, Arnaud being dressed as a CEO with a nice shirt and tie and Sophie being dressed as a "sexy secretary" with a wide neckline blouse revealing her bra. They are immediately attracted to each other.

When it's Arnaud's turn to play “beer pong”, he asks Sophie to be his partner. Sophie is delighted that he has proposed this and joins him immediately. While they are playing, Sophie starts flirting with Arnaud, whom she likes very much.

They have a great time, win a couple of games and realize that they make a good team. When they end up losing, they are replaced by another team. Sophie walks towards Arnaud, touches his arm in a playful way and thanks him for the great time she is having. Arnaud sees his chance, grabs her by the waist to pull her to him, hugs her and lets his hand wander over her buttocks. This makes Sophie feel uncomfortable and Arnaud realizes that Sophie does not seem to be responding warmly. To get out of the situation, Sophie tells Arnaud that she will go looking for her friends, she goes back to the room they entered first and has a beer again. No one is in the room when Sophie enters. Arnaud follows her and notices that they are the only people in the room. He approaches her from behind while she is opening her beer.

Arnaud puts his arms around her and starts to unbutton her blouse. Surprised, Sophie puts down her beer and pushes Arnaud's hands away. Arnaud is amused, thinking that Sophie wants to make herself wanted. He grabs her by the waist, pulls her to him and starts kissing her. At first, Sophie kisses him back, thinking this quite harmless, until Arnaud starts to unzip her skirt. She immediately tells him to stop, moves back and heads for the door. Arnaud, excited, rushes forward and locks the door saying: "What’s the problem? Are you afraid to have a little fun?” He approaches her again and this time Sophie kisses him first. Arnaud then takes off Sophie's blouse and starts to unhook her bra. After taking off Sophie's bra, Arnaud's hands begin to pull down her already unzipped skirt. Sophie twists and, feeling uncomfortable, gently whispers to Arnaud to stop. Arnaud has a grin on his face, thinks she is excited, and kisses her harder, while pushing her against the wall. While he pulls down his pants, Sophie says no and steps aside. Arnaud doesn't want them to stop having fun, so he grabs her by the arm and pushes her against the wall again. Feeling that her attempt to stop the situation has failed, Sophie freezes and remains silent. Hearing no more protests, Arnaud has sex with Sophie. When Arnaud has finished, Sophie quickly puts her clothes back on and leaves the room without saying anything.
